# Depression and Psychological Trauma: An Overview Integrating Current Research and Specific Evidence of Studies in the Treatment of Depression in Public Mental Health Services in Chile

**DOI:** 10.1155/2014/608671

**Published:** 2014-02-17

**Authors:** Verónica Vitriol, Alfredo Cancino, Kristina Weil, Carolina Salgado, Maria Andrea Asenjo, Soledad Potthoff

**Affiliations:** ^1^Department of Psychiatry, Medical School, Universidad de Talca, Chile; ^2^Mental Unit Hospital de Curicó, Curicó, Chile; ^3^Communal Mental Health Program, Communal Health Department of Curicó, Chile; ^4^Mental Health Formative Practices, Medical School, Universidad de Talca, Chile; ^5^Department of Psychiatry, Medical School, Universidad de los Andes, Santiago de Chile, Chile; ^6^Department of Psychiatry, Universidad de Talca, Chile; ^7^Mental Health Unit, Hospital de Talca, Chile; ^8^Mental Health Unit, CESFAM Curicó-Centro, Curicó, Chile

## Abstract

In the last two decades, different research has demonstrated the high prevalence of childhood trauma, including sexual abuse, among depressive women. These findings are associated with a complex, severe, and chronic psychopathology. This can be explained considering the neurobiological changes secondary to early trauma that can provoke a neuroendocrine failure to compensate in response to challenge. It suggests the existence of a distinguishable clinical-neurobiological subtype of depression as a function of childhood trauma that requires specific treatments. Among women with depression and early trauma receiving treatment in a public mental health service in Chile, it was demonstrated that a brief outpatient intervention (that screened for and focused on childhood trauma and helped patients to understand current psychosocial difficulties as a repetition of past trauma) was effective in reducing psychiatric symptoms and improving interpersonal relationships. However, in this population, this intervention did not prevent posttraumatic stress disorder secondary to the extreme earthquake that occurred in February 2010. Therefore in adults with depression and early trauma, it is necessary to evaluate prolonged multimodal treatments that integrate pharmacotherapy, social support, and interpersonal psychotherapies with trauma focused interventions (specific interventions for specific traumas).

## 1. Introduction

Depression is a major cause of morbidity worldwide [1]. One of the risk factors that is associated with the development of this illness and its increased clinical severity is the exposure to early traumatic events [2].

Standardized clinical classifications such as the Diagnostic and Statistical Manual of Mental Disorders (DSM) and the International Classification Disease (ICD) have postulated a single unipolar depression [3, 4]. Nevertheless, there is clinical and neurobiological evidence that suggests a distinguishable subtype of depression as a function of childhood trauma that ought to require specialized treatments [5].

Most of the approaches related to trauma have considered Posttraumatic stress disorder (PTSD) as the main diagnosis [6]. However, depression is two times more prevalent than PTSD in the general population [7]. Even though both pathologies are very often present in comorbidity [8], the study of depression as a result of trauma exposure is scarce. This area needs more attention, not only in trauma research but also in public mental health policies.

In Chile, like in many other countries, there is a legally mandated program to treat depression in mental health services, but the clinical guidelines do not include specifications regarding the approach to patients with depression and childhood trauma [9, 10].

Since 2002, our team has developed successive research on the prevalence of early trauma in adult patients with depression who are being treated through the primary care and/or secondary specialized care of the Public Health Service in Región del Maule. (The region is located 180 Km south of Santiago, the capital of Chile.)

The objectives of this paper are

1. to summarize the epidemiological and clinical findings in adults with depression and childhood trauma in treatment in the Mental Health Services in Chile,
2. to further understand the risk factors that explain the relation between depression and psychological trauma,
3. to integrate these findings in a model of treatment.

## 2. Epidemiological Aspects 

During the past decade, international studies showed that over 50% of the general population has been exposed to psychological trauma [11, 12]. In USA, 61% of men and 51% of women have been exposed to potentially traumatic events [12, 13]. In Chile, 41.7% of men and 33.2% of women have had this previous reference [14].

While men are more exposed to noninterpersonal trauma, women are seriously affected by interpersonal trauma, of which physical and sexual events are the most common [12–14]. In these cases, polytrauma is more frequent than single trauma [14]. Despite the high prevalence of trauma in the general population, not all exposed persons develop a mental disorder related to the trauma [12]. Female gender, interpersonal trauma, and childhood trauma constitute risk factors associated with the further development of psychopathology [15, 16].

A series of epidemiological studies has demonstrated that childhood abuse is associated with a range of psychiatric disorders in adulthood that includes mood, anxiety, and substance abuse disorders [17].

PTSD is the most studied diagnosis as a result of trauma, including early traumatic experiences [6]. However, there is strong evidence demonstrating that emotional neglect as well as physical and sexual abuse during childhood constitutes risk factors in the development of depression in adulthood [2, 18–20] not only in one depressive episode but in its recurrence [21].

Depression is two times more prevalent than PTSD in the general population, being a major cause of morbidity worldwide. In most countries, the number of people who will suffer from depression during their lives falls within an 8%–12% range [1]. According to the World Health Organization, in 2004 unipolar depressive disorders were ranked as the third leading cause of the global burden of disease and they will move to the first place by 2030 [22].

In the past decade, in Chile, according to an epidemiologic study, using the Composite Diagnostic International Interview (CIDI), the prevalence of major depression and PTSD was 9% and 4.4%, respectively [23]. Both pathologies were affecting twice the number of women than men. Recent studies have found that 17.7% of the general population has had depressive symptoms in the last year, with 25.5% occurring just in women [24].

It is also known that one-third of Chilean adults have been exposed at least to one psychological trauma during their lives [14] and, according to a recent UNICEF report, 71% of children of 14 years old referred to having experienced some form of violence by their parents. Of them, 25.9% experienced severe physical violence and 8.7% sexual abuse [25].

Though depression, since 2006, was incorporated in the then newly mandated law, Garantías Explícitas en Salud (GES), and government economic support was included to treat this pathology [26], the importance of early trauma on depression has not been sufficiently studied in the Chilean population [27]. In 2001, when the national program to treat depression had been promoted, the study conducted by Alvarado found that risk factors associated with depression among women being treated by primary care in Santiago of Chile were one previous depressive episode, social isolation and low social support, severe family dysfunction, and a family history of suicide or attempts of suicide. Of them, low social support and suicide among relatives were statistically associated with more severity [27]. Nevertheless, in this study, record of childhood trauma was not investigated as a risk factor for developing depression.

Since 2002, in Región del Maule, we have focused our research on the prevalence of early trauma in adult patients with depression which in treatment at the primary care and secondary specialized care [28–30]. All these studies have used the Marshall scale [31]. This scale is a screening that inquires whether an individual has memories of having one or more of the following traumatic experiences before the age of 15: traumatic separation from a parent or caregiver, alcohol or drug abuse by a family member, physical violence between parents or caregivers, systematic punishment by parent or caregiver, physical injury associated with punishment, and/or forced sexual contact with a relative or a nonrelative. In these studies, which were conducted in different clinical samples, we founded a range of prevalence between 52.1% and 87.5% of psychiatric patients who recalled at least one of the childhood traumatic events [28–30] (Table 1).

Two of these investigations were specifically developed in patients with depression. One of them studied 442 women with mild and moderate depression treated in the primary care program [29]. The other one studied 130 women with severe depression treated in the secondary specialized care program [28]. In both studies, according to the Marshall scale, the most frequently recalled event was alcohol or drug abuse by a family member, which was found to be 48.2% and 67.7%, respectively. The second most frequent recalled event was a traumatic separation from a parent or caregiver and was determined to be between 40% and 50% of the patients. The third most frequent being the systematic punishment by a parent or a caregiver and was reported in 36% of primary care patients and 61% of secondary specialized care patients. Furthermore, 38.8% of the primary care group and 42% of the secondary care group with severe depression mentioned they had experienced sexual abuse during childhood.

In the previously referenced study involving 130 women with severe depression, using the CIDI, we found the prevalence of PTSD to be 49.2% [32]. When the prevalence of PTSD was considered in relation to the number of affirmative answers on the Marshall scale, the prevalence was an increasing function of the number of childhood traumatic events reported by the patients [32]. When comparing patients with three or more childhood traumatic events in relation to those who had less than three of these events, the application of CIDI indicated a higher average of exposure to traumatic events during adulthood (3.87 versus 2.15; *t* = 5.014; *P* < 0.001) and an earlier age of exposure to the most traumatic event (17 versus 27 years old). Likewise, in these patients it was found that the accidents, sexual harassment, rape, or physical aggression was the most significant CIDI event associated with their pathology [32].

In 2010, in another study conducted in the secondary care program, we observed the prevalence of posttraumatic symptoms among 75 women in treatment for severe depression who had experienced the megaearthquake on F-27. After six months 53% of the patients demonstrated PTSD related to the disaster [33], twice that which occurred in the general population exposed to this disaster [34]. Early trauma and acute stress disorder were the risk factors associated with the postcatastrophe PTSD in these patients [33].

These results indicate the high prevalence of childhood trauma, including emotional, physical, and sexual abuse, in patients who are in treatment for depression at public mental health services in Region del Maule, Chile [28, 29]. These findings are associated with greater severity of symptoms and comorbidity. In accordance to these results, it should be necessary to actively question patients with depressive symptoms about their childhood trauma history, in order to determine comorbidity and, hence, to design specific strategies for treatment.

## 3. Neurobiological Factors That Link Childhood Trauma to Adult Depression

There is strong evidence that childhood trauma is associated with a several neurobiological findings, which may configure the diathesis for adulthood psychopathology [35].

Childhood trauma is associated with sensitization of the neuroendocrine and autonomic stress response, glucocorticoid resistance, increased central corticotropin-releasing factor (CRF) activity, immune activation, and reduced hippocampal volume. A major focus of investigation in this area has been the role of the hypothalamic-pituitary-adrenal (HPA) axis, both as a marker of the stress response and as a mediator of additional downstream pathophysiologic changes. The HPA axis functions in close concert with the Locus Coeruleus-Norepinephrine (LC-NE) system, which is involved in extensive reciprocal innervation of regions throughout the central nervous system (CNS) [36, 37].

The dysregulation of HPA axis also involves changes in the pituitary responsiveness to CRF stimulation suggesting changes of CRF receptors due to alterations in the activity of the paraventricular nucleus (PVN-) median eminence CRF circuit. This impairment of physiological adjustment is shown by an increased and blunted adrenocorticotropic hormone (ACTH) response to corticotropin releasing hormone (CRH) stimulation [37].

In clinical samples, Heim et al. report that women with a history of child abuse with major depression showed significantly increased cortisol responses to psychological stress as compared with healthy control subjects and abused women without depression. In (CRH) stimulation tests among these women, those with a history of childhood abuse without depression exhibited increased ACTH responses along with normal-to-decreased cortisol responses. In contrast, women with a history of childhood abuse with comorbid major depression exhibited blunted ACTH responses, likely due to chronic overexposure of the pituitary to CRH. These findings suggest that there may be an initial sensitization of the stress hormone system during early life adversity, representing a biological vulnerability for the development of depression and anxiety disorders in later life [38].

In another study, these same investigators examined 66 women divided into four groups: (1) healthy without early life stress (H) (2) history of childhood abuse without major depression (CA) (3) history of childhood abuse and current major depressive disorder (CAMDD) (4) current major depressive disorder but no early life stress (MDD). Plasma ACTH and cortisol responses to ovine CRH 1 *μ*g/kg and plasma cortisol responses to ACTH 250 *μ*g were measured. In comparison with the H group, CA subjects exhibited enhanced ACTH responses to CRH administration, whereas the CAMDD and MDD groups demonstrated blunted ACTH responses; cortisol responses to CRH were blunted in the CAMDD and CA groups relative to the H group. In the ACTH stimulation test, the CA group exhibited lower baseline and stimulated plasma cortisol concentrations than the H subjects, whereas the CAMDD group showed lower baseline cortisol levels only. These findings also support the etiological hypothesis that depression and anxiety in these women are related to stress exposure later in life resulting in hypersecretion of CRH and downregulation of adenohypophyseal CRH receptors [39].

Another neurobiological mechanism altered by early trauma is in relation to the neuropeptide oxytocin (OT). This neuropeptide has an important role in mediating social affiliation, mother-child attachment, social support, and trust. Further, OT has stress-protective effects and decreases amygdala reactivity in humans [40]. Evidence shows a decreased OT level in central nervous system in patients with history of childhood trauma. These results support the hypothesis that early adverse experience may interfere with the development of brain systems implicated in social attachment, which may then lead to decreased resilience against stress and anxiety [41].

The most available evidence suggests that the pathways affected in subjects with a history of childhood maltreatment are predominantly in frontolimbic networks, including medial prefrontal cortex (mPFC), orbitofrontal cortex (OFC), anterior cingulate cortex (ACC), hippocampus, and amygdala. These pathways are involved in emotion and motivation processing, as well as the control of aggression. Disruption to these pathways in abused subjects may, therefore, underlie the observed deficits in emotion and reward processing as well as excessive aggression or violent behavior. Deficits in these frontolimbic networks may be due to the vulnerability of the frontal lobe to stress effects due to the fact that it has a high density of glucocorticoid receptors and dopaminergic projections that are stress susceptible [42].

It is very important to consider sex differences in the neurobiological findings associated with childhood trauma [43]. It is well known that sex steroids interact with other neurotransmitter systems involved in the stress response, such as the serotonin system. Estrogen receptor mRNA has been localized in parvocellular CRH neurons of the paraventricular nucleus of hypothalamus (PVN) and there is evidence for an estrogen-responsive portion of the promoter region of the human CRF gene, which confers estrogen enhancement of CRF expression in CV-1 transfected cells [44]. Progesteron has also been implicated in modulating these systems involved in stress response. However, it has been observed that women exhibit greater magnitude and duration of HPA axis responses to stress than men independent of acute gonadal steroid effects. Other factors that might determine sex differences in the stress response include genomic differences, organizational differences in brain structures, or developmentally programmed effects of gonadal steroids. In addition, sex steroids play a role in lifelong structural plasticity of several brain regions, including areas involved in stress responsiveness, like hippocampus and amygdala [45].

The effects of childhood trauma on later vulnerability to stress and disease are also moderated by genotype [46]. The serotonin transporter gene 5 HTTLPR significantly moderates the effects of stressful life events on depression. The carrier L/L is resilient to the depressogenic effect of life stress so the s/s predisposes to depression [47]. Depression risk as a function of child mistreatment and 5 HTTLPR status is further modified by social support and a polymorphism in the brain-derived neurotrophic factor (BDNF) [48].

Another gene involved in the relation between childhood trauma and adult depression is the CRH Receptor-1 (CRHR-1) gene. This gene moderates the effects of specific types of childhood trauma on depression and explains the relation between female gender, sexual abuse, and adult depression [49].

These neurobiological changes secondary to early trauma can provoke a neuroendocrine failure to compensate the response to challenge and reflect a risk to develop depression in response to stress in adult life. It suggests the existence of biologically distinguishable subtype of depression as a function of childhood trauma [38].

## 4. Clinical Considerations

Childhood trauma promotes the development of a spectrum of psychiatric and medical disorders [17]. This includes anxiety disorders [50], somatization disorder [30], PTSD [6], personality disorders [51], depression [52], alcohol and drugs abuse [53], eating disorders [54], chronic fatigue syndrome [55], fibromyalgia [56], functional gastrointestinal disorders [57], and cardiovascular diseases [58].

An important comorbidity related to childhood trauma is the association with anxiety, depressive, and somatoform symptoms [59]. These comorbidities often present in general medical patients are associated with more expensive costs in health resources [60–62]. In our country, among 505 medical inpatients in four general hospitals, we found a high exposure to childhood trauma, which was associated with PTSD, somatization disorder, depression, and personality disorder [30].

As previously mentioned, depressive disorders and PTSD occur very often in comorbidity [63, 64]. They share common risk factors such as female gender and history of childhood trauma [65]. Each one is a factor of vulnerability for the development of the other one [63]. In addition, the PTSD-depression comorbidity has been associated with increased clinical severity, suicidality, chronicity, and greater spending on health resources [66–69]. Even though, this pathology is underdiagnosed in clinical patients [70].

There is evidence that, in patients with a history of childhood trauma, PTSD is more complex, correlated with personality disorders, particularly superimposed on borderline personality disorder [71]. These patients often present anxiety and depressive symptoms, alterations in emotional regulation, and revictimization phenomena [72]. Considering these findings it is postulated that patients with early trauma should receive a special category “complex PTSD,” joining axes I and II of DSM classification, and they should be considered differently from borderline patients [73].

Patients with depression and early trauma also show major symptomatic severity, increased risk of suicidality, and interpersonal difficulties [74–76]. In the same sample of 130 women evaluated in Curicó, it was shown, using several scales to evaluate symptomatic severity, that women with severe depression and early trauma had significant severity in depressive symptoms, PTSD symptoms, and interpersonal difficulties (Table 2) [77]. In this study, patients with early trauma had twice the number of suicide attempts than those severely depressed women without early trauma history [77]. We did not evaluate personality disorder but these findings suggest that these patients have the same clinical picture as described in patients with complex PTSD.

Another relevant clinical aspect described in women with a history of sexual abuse is the revictimization phenomenon throughout their lives, including transgenerational transmission of the psychological trauma [79, 80]. Most of women with previous sexual abuse events have a history of recurrent rape and traumatization. They also have repressed and silent suffering in all aspects of their lives and the abuse is still seriously affecting them and their loved ones including their children [81]. These aspects were also observed by us in our patients [82, 83]. The patients had been using the public health service to a great extent, but not getting adequate help and indeed many times they had been mistreated by the medical staff (referred on, postponed, or rejected) [84]. The traumatic background is not routinely inquired about when these patients are interviewed when they seek medical help [85].

In brief, in our region, we correlated that patients with depression and childhood trauma show a complex, severe, and chronic psychopathology. These findings suggest the existence of a clinical distinguishable subtype of depression as a function of childhood trauma that requires specific treatments.

## 5. Treatment Approaches 

In spite of evidence that patient with childhood trauma present a neurobiological clinical subtype of depression, its management is not recommended enough in clinical guidelines [10].

Research examining the effects of childhood trauma on treatment response in depression is limited. One study found that patients with chronic depression and early trauma respond better to psychotherapy alone versus pharmacotherapy [86]. Another three studies showed that nonresponsiveness to antidepressant medication was associated with childhood abuse in both outpatients and inpatients settings [87–89]. There is a need to develop modes of treatment for patients with depression and having a history of childhood abuse and furthermore a need to evaluate their effectiveness.

Considering the psychotherapies with empirical evidence to treat depression, interpersonal psychotherapy (IPT) is one of the most relevant at this point. IPT is a time-limited psychotherapy that focuses on social and interpersonal problems in the patient's current life, for understanding and treating symptoms [90].

There is evidence that patients with early trauma often seek care due to interpersonal problems, especially re-victimization phenomena [91, 92]. These interpersonal difficulties can be understood considering the neurobiological consequences of psychological trauma [93]. But also, these difficulties can be understood from the psychoanalysis paradigm, considering the concept of *compulsion to repeat the trauma* [94–96].

Linking the evidence of IPT in treating depression and the compulsion to repeat the trauma to understand interpersonal difficulties among patients with early trauma, we organized a model of intervention that we call IMT (interpersonal model to treat patients with depression and childhood trauma history) [97].

It is relevant to consider that, in the first interview, early trauma and interpersonal difficulties in patients with depression are inquired about. Then, this model provides a focus on a current dysfunctional interactional pattern associated with depression, in which aggression and victimization are at the center. Such a pattern is understood as a repetition of childhood traumatic experiences. The intervention focuses on developing a cognitive understanding of personal characteristics and behavior allowing the repetition of traumatic experiences in the present. The goal of the intervention is to discriminate the present from the past, avoid the revictimization, and promote the devictimization [97].

During 2006, we evaluated this model associated with pharmacotherapy in a three-month structured intervention, comparing 44 women treated by IMT with 43 women treated as usual. They were evaluated at baseline, at the third and sixth months, with the Hamilton scale for depression (Ham-D), the Lambert questionnaire (OQ-45.2) (which includes the social and interpersonal subscales), and the eight-item questionnaire (PTO-8) to evaluate PTSD symptoms. At three months, significant differences were found in favor of the intervention group in Ham-D scores (*P* < 0.01) and in OQ-45.2 scores (*P* < 0.05). In OQ-45 social subscale, we only found clinical differences in favor of IMT at the sixth month. In OQ-45 interpersonal subscale, only the IMT group obtained significant improvement. In PTO-8 there were no significant differences between both groups (Table 3) [78].

If we consider remission as the goal of the treatment of depression, only 22% of the intervention group attained the goal (versus 5.6% in control group) [78].

We demonstrated that the proposed intervention was more effective than the usual treatment in patients with depression and childhood trauma history. However, the intervention group persisted with symptomatic and dysfunctional indicators at the end of the observation. These findings might be explained through brevity of intervention and suggest more evidence that these patients need prolonged multimodal treatments [98].

But, what kind of prolonged multimodal treatment do these patients need? A prolonged interpersonal model [99–101] or an interpersonal model integrated with a trauma focused model [102]? Why do we ask this?

In 2010, we observed the evolution of 75 women in treatment for depression who experienced the megaearthquake in Chile, 75% of them had history of childhood abuse. Although they continued with an intensive pharmacological and interpersonal support treatment very soon after the disaster, six months later 53% of them showed PTSD according to ICD. The intervention helped the women by the sixth month to improve their clinical global impression, thus, avoiding hospitalization and preventing suicide attempts [103]. These results suggest that these patients would require specific trauma focused interventions immediately after a disaster of this magnitude to prevent PTSD [103].

So, we need to investigate not only prolonged interpersonal treatment in patients with depression and a record of psychological trauma [99–101] but also the effectiveness of specific trauma focused interventions in patients who seek care and the principal diagnosis is depression, mainly when there is a comorbidity with PTSD [102], for example, using exposure therapy in a second step [104, 105] or prescribing other medication like propranolol [106].

In summary, patients with depression and history of interpersonal psychological trauma since childhood have a complex profile, so they need to be structured in a program that addresses its many complexities and not only the depressive symptoms.

## Figures and Tables

**Table 1:** Prevalences of childhood trauma among general morbidity patients and psychiatric patients in Chile.

Place/authors	Sample	At least one item in Marshall scale*	% sexual abuse
4 General hospitals (Weil et al., 2004 [30])	505 general medicine patients, both genders (263 men and 242 women)	54.7%	6.8%
Hospital Curico (Vitriol et al., 2006 [28])	130 women ingressed for severe depression	87.5%	42%
CES Curico-Centro (Cancino and Asenjo, 2006 [29])	510 patients (442 women and 68 men) ingressed for nonsevere depression	84%	38.8%
Hospital TALCA (Salgado, Potthoff, 2012) unpublished observation presented at the 21st World Congress for Social Psychiatry, Lisbon, Portugal, 2013	593 psychiatric patients (453 women and 140 men)	52.1%	35.2%

*Marshall scale [31] is a screening that inquires whether an individual has memories of having one or more of the following traumatic experiences before the age of 15: traumatic separation from a parent or caregiver, alcohol or drug abuse by a family member, physical violence between parents or caregivers, systematic punishment by parent or caregiver, physical injury associated with punishment, and forced sexual contact with a relative or a nonrelative.

**Table 2:** Average scores in Hamilton, OQ 45 and PTO 8 between 81 women with severe depression and childhood trauma and 49 women with severe depression and without childhood trauma [77].

Test	Average with trauma	DS	Average without trauma	DS	*T*	*P*
Marshall^1^	4.17	1.34	1.57	1.37	10.32	0.01
Hamilton D^2^	34.67	6.24	24.02	9.57	7.59	0.01
OQ 1 total^3^	115.04	20.0	84.84	31.6	6.58	0.01
OQ symptoms^4^	73.08	12.2	56.57	20.9	5.63	0.01
OQ Interpers. Rel.^5^	24.08	6.59	15.86	8.63	6.005	0.01
OQ 1 Social role^6^	18.02	6.06	12.09	6.61	5.096	0.01
PTO 8^7^	18.00	8.17	8.18	8.85	6.264	0.01

^1^Marshall scale: scores between 0 and 7 points according to childhood trauma events.

^2^Hamilton depression scale: scores between 0 and 63 points with higher scores indicating a greater severity of symptoms.

^3^OQ 45 global scale: scores between 0 and 180 points with higher scores indicating worse function.

^4^OQ 45 symptoms scale: scores between 0 and 100 points with higher scores indicating a greater severity of symptoms.

^5^OQ 45 interpersonal scale: scores between 0 and 44 points with higher scores indicating worse function.

^6^OQ 45 social role scale: scores between 0 and 36 points with higher scores indicating worse function.

^7^PTO 8 scale: scores between 0 and 32 points with higher scores indicating a greater severity of symptoms.

**Table 3:** Symptoms changes over time among 87 severe depressive women with childhood trauma, 44 of them assigned to protocolized interpersonal model of trauma (IMT) versus 43 who received usual treatment. Curicó, Chile [78].

Outcome measures and groups	Admission		Discharge		6 months follow up	
	Mean	(SD)	Mean	(SD)	Mean	(SD)
Hamilton^1^						
Protocol group	34.09	6.2	22.1	8.8*	19.4	8.8**
Control group	34.42	6.7	27.5	9.7	25.0	11.1
OQ45 total^2^						
Protocol group	112.73	19.7	92.7	28.3*	85.8	37.6*
Control group	114.72	22.5	105.4	31.0	102	31.7
OQ45 symptoms^3^						
Protocol group	71.07	12.2	59.1	18.4	53.5	22.8*
Control group	73.37	13.6	65.6	17.0	63.4	18.9
OQ45 Interpers. Rel.^4^						
Protocol group	24.5	6.6	19.0	7.7	18.7	10.2
Control group	23.1	6.9	22.6	11.1	21.6	8.7
OQ45 social^5^						
Protocol group	17.4	5.5	14.5	6.8	13.5	8.2*
Control group	18.3	6.7	17.1	7.9	16.8	7.1
Posttraumatic symptoms scale (PTO-8)^6^						
Protocol group	16.6	7.6	12.6	7.8	11.7	7.9
Control group	19.1	8.4	14.9	8.1	15.0	7.8

^1^Hamilton: scores between 0 to 63 points with higher scores indicating a greater severity of symptoms.

^2^OQ45 global scale: scores between 0 to 180 points with higher scores indicating worse function.

^3^OQ45 symptomatic scale: scores between 0 to 100 points with higher scores indicating a greater severity of symptoms.

^4^OQ45 interpersonal scale: scores 0 to 44 points with higher scores indicating worse function.

^5^OQ45 social role scale: scores between 0 to 36 points with higher scores indicating worse function.

^6^PTO 8 scale: scores between 0 to 32 points with higher scores indicating a greater severity of symptoms.

* < 0.005, ** < 0.001.
